# A novel multidomain acyl-CoA carboxylase in *Saccharopolyspora erythraea* provides malonyl-CoA for *de nov*o fatty acid biosynthesis

**DOI:** 10.1038/s41598-019-43223-5

**Published:** 2019-04-30

**Authors:** Andrea L. Livieri, Laura Navone, Esteban Marcellin, Hugo Gramajo, Eduardo Rodriguez

**Affiliations:** 1 Instituto de Biología Molecular y Celular de Rosario, Facultad de Ciencias Bioquímicas y Farmacéuticas, Universidad Nacional de Rosario, Rosario, Argentina; 2 Australian Institute for Bioengineering and Nanotechnology, The University of Queensland, Brisbane, Queensland Australia; 3 Present Address: Molecular Biology & Industrial Biotechnology, Science and Engineering Faculty, Queensland University of Technology, Brisbane, Queensland Australia

**Keywords:** Microbiology, Evolution

## Abstract

Acetyl-CoA carboxylases (ACCs) are enzyme complexes generally composed of three catalytic domains and distributed in all organisms. In prokaryotes and plastids of most plants, these domains are encoded in distinct subunits forming heteromeric complexes. Distinctively, cytosolic ACCs from eukaryotes and plastids of graminaceous monocots, are organized in a single multidomain polypeptide. Until now, no multidomain ACCs had been discovered in bacteria. Here, we show that a putative multidomain ACC in *Saccharopolyspora erythraea* is encoded by the *sace_4237* gene, representing the first prokaryotic ACC homodimeric multidomain complex described. The SACE_4237 complex has both acetyl-CoA and propionyl-CoA carboxylase activities. Importantly, we demonstrate that *sace_4237* is essential for *S. erythraea* survival as determined by the construction of a *sace_4237* conditional mutant. Altogether, our results show that this prokaryotic homodimeric multidomain ACC provides malonyl-CoA for *de novo* fatty acid biosynthesis. Furthermore, the data presented here suggests that evolution of these enzyme complexes, from single domain subunits to eukaryotic multidomain ACCs, occurred in bacteria through domain fusion.

## Introduction

Acetyl-CoA carboxylases (ACC)s catalyse the carboxylation of acetyl-CoA to malonyl-CoA (E.C. 6.4.1.2), the first step in fatty acid biosynthesis in prokaryotes and eukaryotes^1,2^. Carboxylation of acetyl-CoA to malonyl-CoA is the rate-limiting step in *de novo* fatty-acid biosynthesis and ACCs have long been used as targets to control metabolic disorders such as obesity, metabolic syndrome and infectious diseases^2,3^. Recently, up-regulation of ACC was found in human cancerogenic tumors, suggesting this enzyme as a potential target for the treatment of cancer^4^. In archaea, ACCs are part of the 3-hydroxypropionate pathway, involved in autotrophic carbon fixation^5^. ACCs belong to the biotin-dependent carboxylase protein family. The two-step reaction mechanism of ACCs involves an ATP-dependent formation of carboxybiotin followed by the transfer of the carboxyl moiety to acetyl-CoA. Both steps are performed by three main functional components, a biotin carboxyl carrier protein (BCCP), a biotin carboxylase domain (BC) and a carboxyl transferase domain (CT). Domain arrangement varies amongst different biotin-dependent carboxylases and from one organism to another^1^. Structural studies have described new non-catalytic domains^6,7^.

In bacteria, the ACC model derives from the *Escherichia coli* complex. Cronan *et al*. (1972) described the role of ACCs in fatty acid biosynthesis by isolating thermosensitive *E. coli* mutants capable of growing at restricted temperatures only when the media was supplemented with saturated and unsaturated fatty acids^8^. Further characterization of the conditional mutants allowed for the identification of four genes encoding different components of the multi-subunit ACC from *E. coli*, which later became a “model bacterial ACC”^9,10^. The *E. coli* ACC contains three domains encoded in four proteins: the BC domain, the BCCP component and two independent peptides (α and ß) that form the functional CT domain.

More recently, various studies have described distinct ACC arrangements in actinomycetes, including *Streptomyces, Corynebacterium* and *Mycobacterium spp*., which differ from the *E. coli* configuration^11–13^ and disproves the universal ACC *E. coli* model in bacteria. For example, the actinobacteria ACCs are heteromeric multisubunit complexes formed by two main subunits, the α subunit containing the BC and BCCP domains, and the β subunit containing the CT domain. Distinctly, these enzymes are able to carboxylate acetyl-CoA and propionyl-CoA and are referred to as acyl-CoA carboxylases. Acyl-CoA carboxylases were first studied in *Streptomyces coelicolor* through the construction of a conditional mutant of the gene encoding AccB, the CT domain of the ACC complex. Throughout this study, the essential role of the enzyme for bacterial growth, as well as its role in actinorhodin polyketide production, was demonstrated^11^. Further studies in mycolic acid-containing bacteria such as *Mycobacterium tuberculosis* and *Corynebacterium glutamicum* allowed identifying the components of others essential ACC complexes^13–15^.

**Figure 1:**
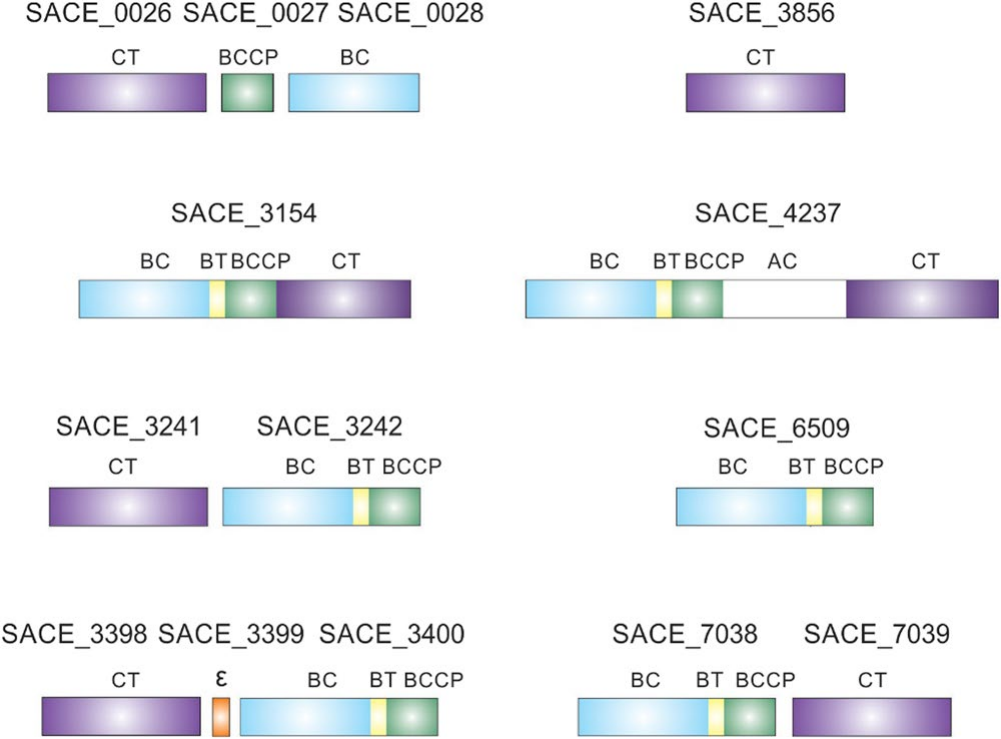
Schematic representation of acyl-CoA carboxylases found in *S. erythraea*. Indicated domains: BC, biotin carboxylase; BCCP, biotin carboxyl carrier protein; CT, carboxyltransferase; Ɛ, epsilon subunit; AC, ACC central; BT, domain that mediates BC–CT interactions.

Strikingly, a large group of actinobacteria do not contain orthologues to the essential CT domain found in *S. coelicolor*, *C. glutamicum* or *M. tuberculosis;* neither to the multisubunit ACC from *E. coli*. These distinctions make predictions difficult in these microorganisms. For example in *Saccharopolyspora erythraea*, the producer of erythromycin A and flaviolin, a vast array of putative biotin-dependent carboxylases are annotated in the genome, including a putative multidomain carboxylase enzyme^16–18^ (Fig. 1).

Here, we characterised SACE_4237 from *S. erythraea*, previously annotated as a putative multidomain carboxylase, using biochemical assays and by constructing a conditional knockout strain. We show that SACE_4237 is an essential multidomain acyl-CoA carboxylase, representing the first of its kind in bacteria. These findings open new avenues to find a possible new ancestor of the eukaryotic multidomain ACC.

## Results

### Phylogenetic analysis

**Figure 2:**
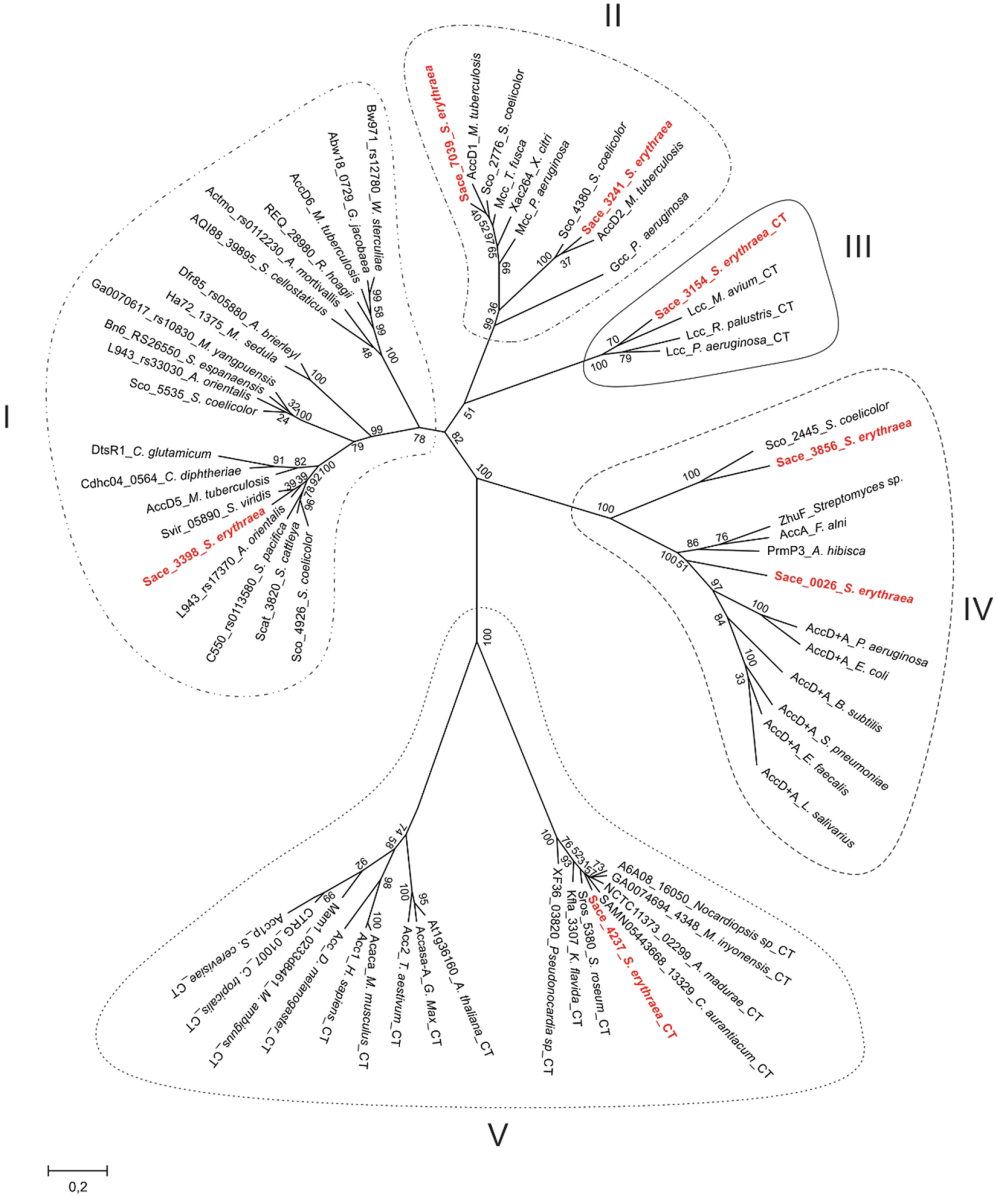
Phylogenetic analysis of CT domains of acyl-CoA carboxylase complexes. The maximum likelihood phylogenetic tree was constructed using the MEGA X software. The bootstap percentage support (1000 replicates) are indicated in the different branches. The tree is organized into distinctive groups (I–V) of closely-related CT domains. The lengths of the branches are proportional to the inferred evolutionary distances. The relative number of substitutions per site is indicated by the bar at the bottom left. The CT domains of *S. erythraea* putative acyl-CoA carboxylases are highlighted in red.

Several studies have shown that the acyl-CoA substrate specificity for the biotin-dependent carboxylase complexes is determined by the CT domain^19^. To predict the substrate specificity of the putative ACCs annotated in the *S. erythraea* NRRL23338 genome, and to identify the essential ACC in this organism (throughout this paper “essential” or “non-essential” refers to the need, or not, of the corresponding enzyme activity for cell viability), we performed a phylogenetic analysis of the CT domains^16^. We also compared them to the CT domains of characterized ACCs from different organisms^11–14,20–22^ using the MEGA X software (Fig. 2)^23^.

The analyses showed that the tree is divided into five groups branching at deep nodes. Furthermore, it showed that the CT domains are grouped with different known carboxylases, suggesting diverse substrate specificity (Fig. 2). Accordingly, group I contains CT domains that correspond to acyl-CoA carboxylases formed by two functional subunits. The group includes essential ACCs from *Streptomyces*^11^, *Corynebacterium* and *Mycobacterium*^12,13^, non-essential propionyl-CoA carboxylase (PCC) complexes from actinobacteria^15^ and CT domains of acyl-CoA carboxylases from archaea^5^. In this group, SACE_3398 clusters with non-essential CT subunits from PCC complexes of other actinobacteria. In addition, earlier studies of acyl-CoA carboxylases in *S. erythraea* have demonstrated that SACE_3398 is part of a non-essential PCC complex^17^.

Group II comprises enzymes that carboxylate the ɣ-carbon of certain α-β unsaturated acids, instead of the α-carbon of saturated acids (e.g. acetyl-CoA, propionyl-CoA). This group contains SACE_3241 and SACE_7039 that are closer related to CT domains of geranyl-CoA and methylcrotonyl-CoA carboxylases, respectively^21^.

The CT domain of SACE_3154 clusters in group III together with other CT domains from long-chain acyl-CoA carboxylases (LCC) that are formed by a unique polypeptide chain and that have been previously described in mycobacteria and *Pseudomonas*^22^. These bacterial LCCs are rare and have been proposed to take role in carbon and nitrogen metabolism.

Group IV includes CT domains from carboxylases belonging to multi-subunit ACCs from Gram-negative bacteria and Firmicutes. The group also comprises CT domains from carboxylase complexes of actinobacteria that are formed by two or three subunits and CT domains that catalyse the reverse reaction; namely they are malonyl-CoA decarboxylase enzymes involved in malonate assimilation^24^. Interestingly, some of these CT subunits are encoded by genes that usually map near or inside polyketide gene clusters. As shown in Fig. 2, SACE_0026 is an ortholog of PrmP3 and it is encoded by the *sace_0026* gene belonging to a putative polyunsaturated fatty acid biosynthetic gene cluster, suggesting that it is not essential for bacterial growth^16^. On the other hand, SACE_3856 is an ortholog of MatA, a malonyl-CoA decarboxylase enzyme from *S. coelicolor* and *Rhizobium trifolli*^24^.

Finally, the CT domain of SACE_4237 emerged in close affiliation with other CT domains of putative multidomain ACCs. All these orthologues clustered (bootstrap support of 100%) with the CT of multidomain ACCs from eukaryotes (group V, Fig. 2). This result suggests that the actinobacterial and the eukaryotic CT domains from group V come from a common ancestor. It is worth mentioning that all actinobacterial CT domains from group V, including SACE_4237, form part of large multidomain proteins of approximately 200 kDa. All have end-to-end homology with the 260–280 kDa eukaryotic ACCs, including the conserved central region. Considering that none of the putative CT domains of *S. eryhtraea* analysed above show evidence of being an essential ACC, led us to hypothesize that SACE_4237 could be the enzyme that provides malonyl-CoA for fatty acid biosynthesis.

### *In vivo* and *in vitro* characterization of SACE_4237

**Figure 3:**
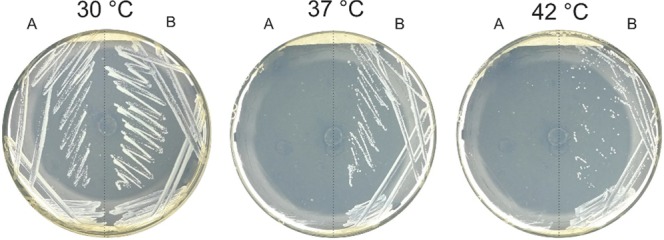
Complementation test for *E. coli* L8 strain. (**A**) L8 strain transformed with pSK bluescript empty-vector. (**B**) L8 strain transformed with pSK-SACE4237 plasmid. LB-agar plates supplemented with 0.5 mM of IPTG grown for 48 h at 30, 37 or 42 °C.

In order to determine the role of SACE_4237 as an ACC, we complemented the *E. coli* L8 strain with SACE_4237^8^. The L8 strain is a temperature-sensitive (*ts*) mutant in the *accB* gene, which causes the deficient biotinylation of the BCCP subunit, resulting in cell death above 37 °C. The *sace_4237* gene was PCR amplified and cloned into the pSK-bluescript vector under the control of the *pLac* promoter, to achieve the plasmid pSK-SACE_4237. This plasmid, as well as the empty vector, were transformed into the *E. coli* L8 strain, respectively. Transformants were tested for growth at different temperatures. As illustrated in Fig. 3, SACE_4237 complemented the growth of L8 mutant strain at 37 °C and 42 °C when IPTG was added. No growth was observed when the mutant strain was transformed with the empty vector, or when the complemented strain was grown in the absence of IPTG (data not shown). The results show that SACE_4237 can complement the deficiency in ACC activity in the *E. coli* L8 strain and suggest that the multidomain SACE_4237 could be the essential ACC in *S. erythraea*.

**Figure 4:**
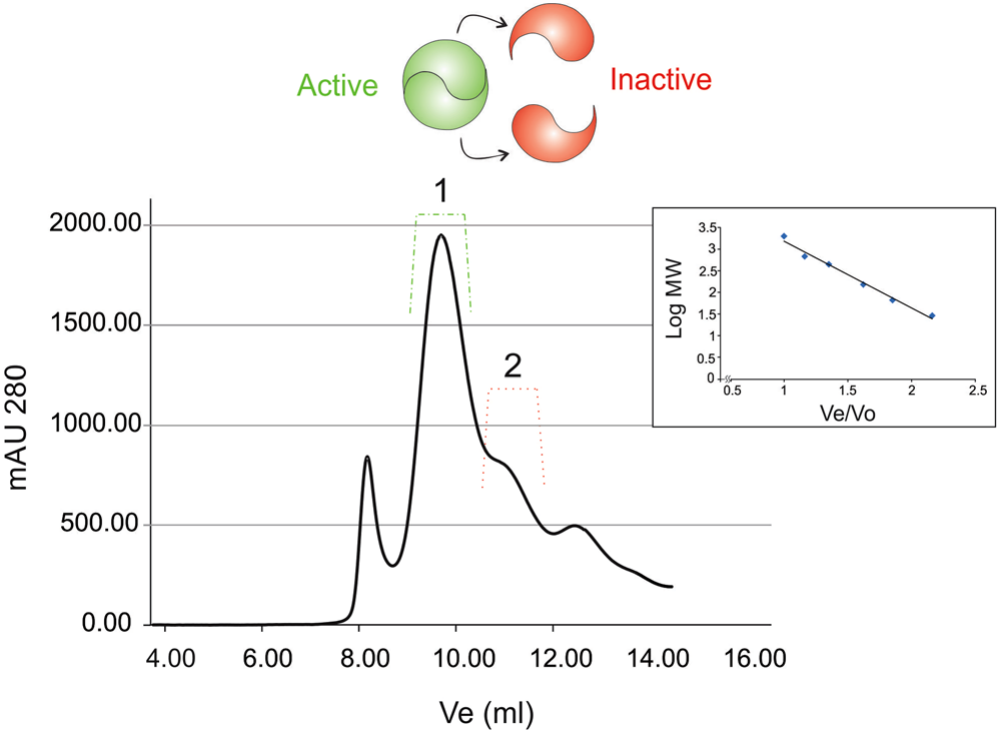
Determination of the oligomeric state of SACE_4237. The purified protein was analyzed by size exclusion chromatography (Superdex 200 10/300 GL, GE). The protein profiles were followed by measuring absorbance at 280 nm (in milli-absorbance units [mAU]). The inset shows the calibration curve obtained with molecular mass standards; MW, molecular weight; Ve, elution volume; Vo, void volume. The calibration curve was used to calculate the molecular weight of the elution peaks 1 and 2, equivalent to 404 and 186.9 kDa, respectively. These peaks correspond to the dimeric and monomeric form of SACE_4237, respectively.

To biochemical characterize the protein SACE_4237, the gene was cloned under the strong *pBAD* promoter, to yield pBAD-SACE_4237. Optimal expression conditions were obtained using *E. coli* protease deficient BL21 strain containing the multicopy plasmid pCY216^25^ which provides a biotin ligase protein to improve SACE_4237 biotinylation. The biochemical studies were carried out by measuring the carboxylation of acetyl-CoA with the purified protein. The two elution peaks from the size exclusion chromatography, corresponding to pure SACE_4237, were assayed for ACC activity (Fig. 4). Carboxylation of acetyl-CoA was only detected with the first elution peak corresponding to a homodimeric complex of approximately 400 kDa, while the monomeric form of the protein (second elution peak) was inactive (Fig. 4; Table 1). Furthermore, carboxylation of propionyl-CoA and palmitoyl-CoA, by SACE_4237 were also tested. SACE_4237 was able to carboxylate propionyl-CoA, while no activity was detected using palmitoyl-CoA as a substrate. The kinetic parameters for the enzyme indicate that SACE_4237 has a slightly higher catalytic efficiency for acetyl-CoA compared to propionyl-CoA (Table 1). Nevertheless, the *K*_m_ for propionyl-CoA was lower than the *K*_*m*_ for acetyl-CoA. The assays confirmed that SACE_4237 behaves as an acyl-CoA carboxylase.

**Table 1:** Kinetic parameters.

Kinetic parameter	Substrate*	
	Acetyl-CoA	Propionyl-CoA
*k*_cat_ (seg^−1^)	0.143 ± 0.004	0.052 ± 0.002
*K*_m_ (mM)	0.168 ± 0.001	0.098 ± 0.001
*V*_max_ (µmoles min^−1^)	0.00043 ± 0.0001	0.00016 ± 0.00001
*k*_cat_/*K*_m_ (seg^−1^ mM^−1^)	0.85 ± 0.03	0.52 ± 0.04

*No activity was detected with palmytoyl-CoA as a substrate.

### The physiological role of SACE_4237

**Figure 5:**
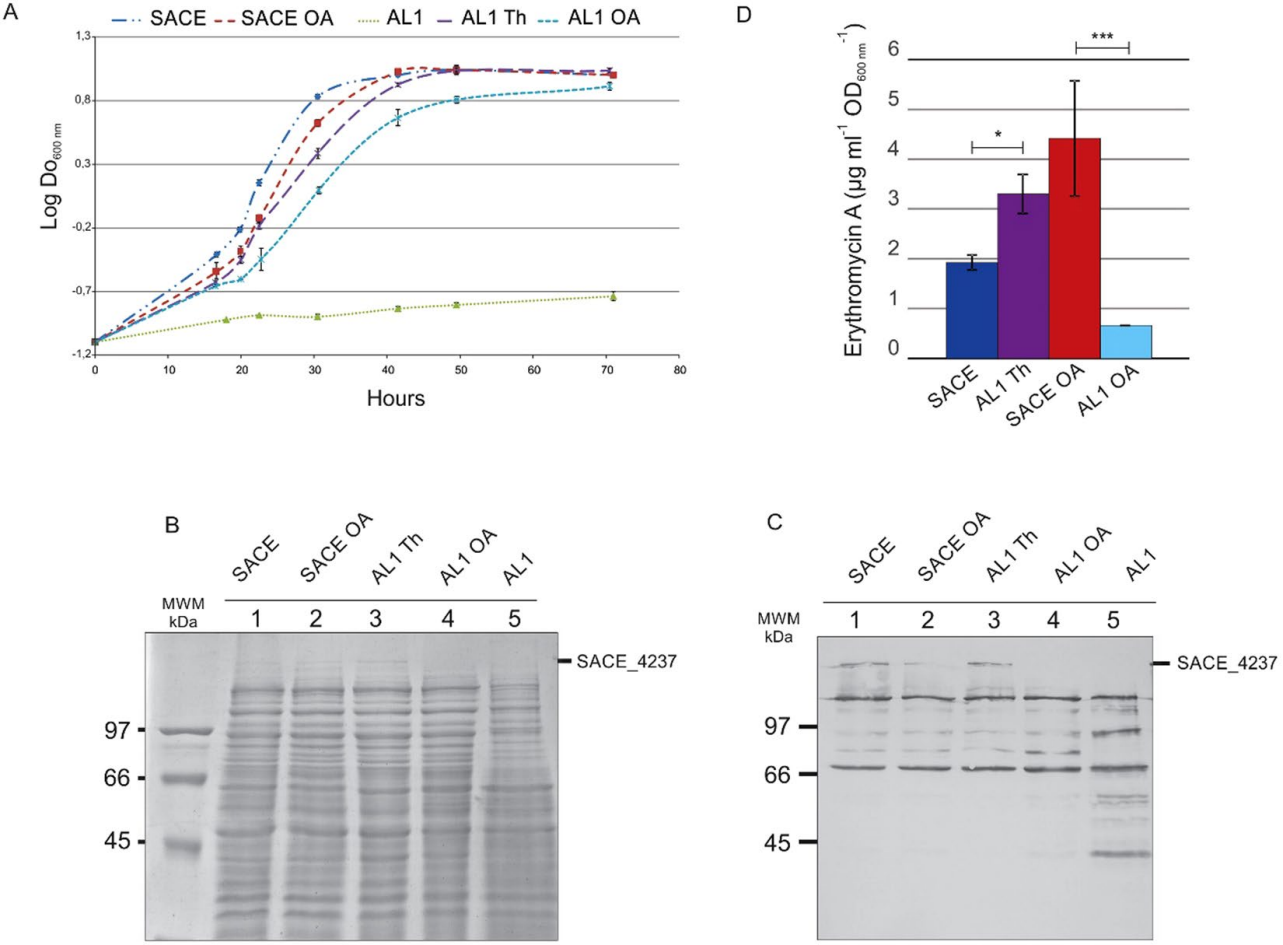
Analysis of *S. erythraea* NRRL23338 and AL1 conditional mutant strains on different growth conditions. (**A**) Growth curves on R5 medium of *S. erythraea* NRRL23338 supplemented or not with oleic acid (OA) and *S. erythraea* AL1 induced or not with Th or supplemented with oleic acid. (**B**) SDS-PAGE of total protein extracts. (**C**) Western-blot of biotinylated proteins. MWM, standard molecular weight markers. (**D**) Erythromicin A production under different growth conditions. SACE, *S. erythraea* NRRL23338; SACE OA, *S. erythraea* NRRL23338 supplemented with oleic acid; AL1 Th, *S. erythraea* AL1 supplemented with Th; AL1 OA, *S. erythraea* AL1 supplemented with oleic acid. Data are represented as mean values ± standard deviation (n = 3). *Statistically significant differences (p ≤ 0.05) between *S. erythraea* NRRL23338 and *S. erythraea* AL1 strains. Analysis was performed using one-way ANOVA.

In order to determine the physiological role of SACE_4237 in *S. erythraea*, we constructed a conditional mutant by exchanging the *sace_4237* native promoter for the thiostrepton (Th) inducible *ptipA* promoter^11^. Homologous recombination was confirmed by PCR and the resulting strain named hereafter AL1. AL1 showed growth dependence to Th in solid and liquid media indicating that the expression of *sace_4237* was essential for cell viability (Fig. 5A). The result suggests that SACE_4237 is the main ACC responsible for making malonyl-CoA for *de novo* fatty acid biosynthesis in *S. erythraea*. To confirm this result, the AL1 strain was conjugated with a plasmid carrying the entire *sace_4237* gene under the permE* promoter, for the constitutive expression of the essential ACC. All kanamycin resistant transconjugants were able to grow in the absence of Th (see Supplementary Fig. S2), implying that the constitutive expression of SACE_4237 was complementing the deficiency of the ACC activity of the conditional AL1 mutant.

To demonstrate that SACE_4237 is the main ACC responsible for making malonyl-CoA for *de novo* fatty acid biosynthesis, the AL1 conditional mutant was grown without Th and supplemented with oleic acid to bypass the absence of fatty acid biosynthesis. The results presented in Fig. 5A show that the exogenous addition of oleic acid enables growth of AL1 in absence of the SACE_4237 expression. Western blot analysis of biotinylated proteins from cell-free extracts of *S. erythraea* NRRL23338 and the AL1 mutant strains grown +/− Th, and supplemented with or without oleic acid, confirmed that SACE_4237 was not expressed in the absence of the inducer Th (Fig. 5B y 5C).

**Figure 6:**
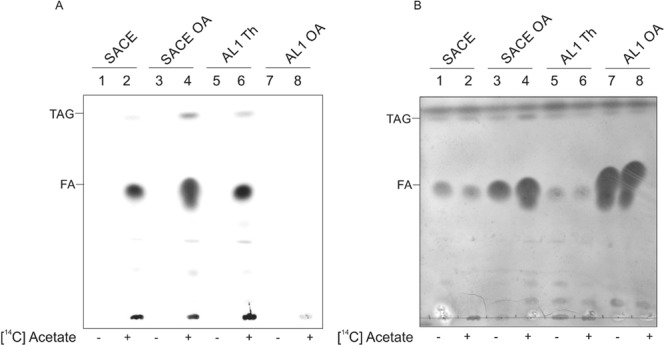
Thin-layer chromatography (TLC) of unlabelled and 14C-labelled lipids from *S. erythraea* NRRL23338 and AL1 conditional mutant strains. Cultures were grown by duplicate in R5 medium to exponential phase (OD_600nm_ = 2) and supplemented with ^14^C-acetate and grown for one additional hour when indicated (lanes 2, 4, 6 and 8). Total lipids extracted from 3 mg of lyophilized cell material with organic solvent and separated on silica gel TLC were developed with hexane-diethylether-acetic acid (50:50:1, v/v/v). (**A**) *De novo* synthesized lipids visualized using a Typhoon FLA 7000 Phosphorimager. Image was overexposed for signal identification. (**B**) Total lipids detected by chemical staining with Cu-phosphoric stain, lipid bands were identified by their co-migration with standards. SACE, *S. erythraea* NRRL23338 strain; SACE OA, *S. erythraea* NRRL23338 strain supplemented with oleic acid; AL1 Th, *S. erythraea* AL1 strain supplemented with Th; AL1 OA, *S. erythraea* AL1 strain supplemented with oleic acid.

To prove that the absence of SACE_4237 expression in the mutant strain had a reduced ACC activity *in vivo* leading to lower levels of malonyl-CoA, we measured the incorporation of ^14^C-acetate into the lipids in *S. erythraea* and the AL1 strains growing +/− Th and +/− oleic acid. As shown in Fig. 6 no incorporation of ^14^C-acetate into *de novo* synthesized fatty acids was observed in strain AL1 confirming the absence of ACC activity. Altogether these results demonstrate that SACE_4237 is the essential ACC in *S. erythraea* responsible for the production of malonyl-CoA, the elongation unit used for *de novo* fatty acids biosynthesis.

Lastly, since SACE_4237 also has PCC activity, we studied the influence of SACE_4237 on erythromycin production under different growth conditions. The parental and AL1 strains were grown for four days in R5 medium in the presence or absence of Th or +/− oleic acid. As shown in Fig. 5D, the parental strain produced more erythromycin when grown in R5 supplemented with oleic acid. On the contrary, the AL1 mutant strain produced more erythromycin in R5 with Th compared to the parental strain; however, reduced antibiotic production was observed in R5 medium supplemented with oleic acid. Our results suggest that SACE_4237 presents PCC activity *in vivo* and provides methylmalonyl-CoA for polyketide production.

## Discussion

ACCs are enzymatic complexes distributed in all domains of life. In bacteria and eukaryotes, ACCs play an essential role in lipid metabolism^1^. Over the years, a vast variety of domain arrangements of ACCs complexes have been established. Up to now, essential ACCs in bacteria were described as heteromeric complexes formed by different subunits. In all bacteria, except for Thermotogales, Spirochaetes and Actinobacteria, ACCs are composed of four subunits^26^. In actinomycetes, such as *Streptomyces* or the mycolic-acid containing bacteria, ACCs are formed by two large subunits, containing all catalytic domains, together with a small non-catalytic Ɛ subunit^11,13^. In other actinobacteria, like the erythromycin producer *S. erythraea*, no orthologue of an essential ACC has been previously characterised. In this work, we identified SACE_4237, an ortholog of the multidomain eukaryotic ACC, and showed that its homodimeric complex behaves as an essential ACC in *S. erythraea*.

*S. erythraea* also has a smaller putative multidomain acyl-CoA carboxylase, SACE_3154. This protein is an orthologue of other carboxylases already described in different groups of bacteria like *Pseudomonas* and *Mycobacterium*^22^. Remarkably, biochemical and structural differences were found between SACE_4237 and the previously described single-chain multidomain carboxylases from *Pseudomonas* and *Mycobacterium*^22^. First, the *Pseudomonas* like multidomain acyl-CoA carboxylase is considered as a long-chain acyl-CoA carboxylase (LCC) showing activity against acyl-CoAs with chain lengths from C_2_ to C_16._ The enzyme shows comparable *k*_cat_ values for all substrates, but the *K*_m_ for palmitoyl-CoA is 350-fold lower than that for acetyl-CoA^22^. Instead, SACE_4237 only shows activity towards short-chain acyl-CoAs (acetyl-CoA and propionyl-CoA) with similar catalytic efficiency. The LCCs are homo hexameric complexes and contain a BC-BCCP domain in the carboxy-terminal region and a CT domain in the amino-terminal region (Fig. 1). On the other hand, active SACE_4237 enzyme forms a homodimer complex, that, in addition to the catalytic domains, also contains a central region only found in eukaryotic ACCs^6,27^. Furthermore, sequence analysis of SACE_4237 central region revealed secondary structure elements that match components present in the crystallographic structure of the *S. cerevisiae* ACC^28^ (see Supplementary Fig. S4). In addition, the phylogenetic analyses of the CT and BC domains (Figs 2 and S3) of SACE_4237 and SACE_3154 show a divergent evolution of these complexes; suggesting that these two enzymes come from two independent fusion events of their domains.

Two isoforms of eukaryotic ACCs have been identified in mammalian cells, an essential cytoplasmic form that provides malonyl-CoA for fatty acid biosynthesis, and a mitochondrial form involved in the regulation of fatty acid oxidation^29^. Both mammalian cytoplasmic and mitochondrial carboxylases form homodimer active complexes that are regulated transcriptionally and post-translationally by different factors^29^. For example, the oligomeric state and kinetic activation of the enzyme activity are modulated by citrate and inactivated by phosphorylation^29,30^. SACE_4237 is also active in a dimeric state; however, our *in vitro* experiments demonstrated that citrate is not required neither for oligomerization nor enzymatic activity. Proteomic analyses carried out by Licona-Casani *et al*.^31^ showed that SACE_4237 is phosphorylated during the metabolic switch, a brief period of growth arrest preceding biosynthesis of secondary metabolites. Thus, we hypothesise that SACE_4237 activity could be modulated by phosphorylation. In addition, the enzyme complex also showed a relaxed substrate specificity, being able to carboxylate acetyl-CoA and propionyl-CoA with similar catalytic efficiency. This characteristic is shared by other heteromeric acyl-CoA carboxylases from actinomycetes^11^. In *S. erythrae*a, the PCC activity of SACE_4237 could be involved in providing methylmalonyl-CoA for erythromycin biosynthesis. In this regard, the *sace_4237* conditional mutant strain AL1 grown in the presence of oleic acid showed reduced erythromycin production compared to the parental strain (Fig. 5), suggesting that SACE_4237 also supplies methylmalonyl-CoA for erythromycin biosynthesis under the condition tested. In previous studies, a *sace_3398* deficient mutant showed reduced PCC activity, but the mutation did not have detrimental effects on erythromycin production^17^. The overlapping activities of two PCC complexes make it difficult to predict their physiological effect on secondary metabolisms. Therefore, each of them should be carefully evaluated to confirm their effect on erythromycin production.

Orthologues of SACE_4237 are present in other actinobacteria, including important secondary metabolites producers, like *Micromonosporaceae*, *Nocardioidaceae*, *Pseudonocardiaceae*, *Nocardiopsaceae*, *Streptosporangiaceae*, *Thermomonosporaceae*, *Cryptosporangiaceae*, *Kineosporiaceae*. Thus, our results highlight the importance of characterizing this new family of homodimeric multidomain acyl-CoA carboxylases for biotechnological purposes.

Evolution of multifunctional polypeptides has been proposed to occur by duplication, fusion and/or recombination of small monofunctional units^32^. Regarding eukaryotic ACCs, Lombard and Moreira^26^ proposed that these complexes evolved by the fusion of two unit polypeptides bearing the BC-BCCP domains and the CT domain. Phylogenetic studies conducted in this work provide new insights into the evolutionary origins of homodimeric eukaryotic ACCs. Our studies indicate that the CT domains of homodimeric acyl-CoA carboxylases from actinobacteria and eukaryotes shared a common ancestor not previously reported (Fig. 2). In addition, the phylogenetic analysis of the BC domains of these enzyme complexes indicates the same distribution observed for the corresponding CT domains (see Supplementary Fig. S4). The presence of a homologous central domain in all multidomain ACCs complexes, their active homodimeric form, and the phylogenetic inferences here described, lead us to propose a new evolutionary theory where the occurrence of a gene fusion event resulted in an “eukaryotic-type” ACC among actinobacteria or in an immediate actinobacterial ancestor. Two alternative scenarios can be foreseen, an actinobacterial organism bearing such fusion evolved into the precursor of all eukaryotes^33^ or, the genes encoding for the multidomain ACC could have been laterally transferred from this prokaryotic organism to the last common ancestor of the eukaryotes. Regardless of the mechanism that leads to the ACCs of present-day eukaryotes, we propose that the term “actinobacterial homodimeric ACCs” should replace the current “eukaryotic-like ACCs” designation.

## Methods

### Bacterial Strains, Culture, and Transformation Conditions

*S. erythraea* strains (see Supplementary Table S1) were grown either on solid or liquid R5 medium (without sucrose) at 30 °C and supplemented when needed with the followings antibiotics: 150 µg ml^−1^ apramycin (Am), 10 µg ml^−1^ thiostrepton (Th) and/or 50 µg ml^−1^ kanamycin (Km)^34^. For conjugation experiments, *E. coli* strain ET12567 harbouring pUZ8002^35^ and a movilisable plasmid were mixed with *S. erythraea* spores and plated on ISP4 medium. After incubation at 30 °C for 16 hours, the plates were then overlaid with Th (300 µg per plate), Am (4.5 mg per plate) or Km (1.5 mg per plate). Fatty acid supplementation studies were performed on R5 liquid medium supplemented with oleic acid 0.01% (v/v), Brij 58 0.075% (v/v) and appropriated antibiotics when needed. All *E. coli* strains (see Supplementary Table S1) were grown in LB media and transformed following standard protocols. Transformants were selected on media supplemented with the appropriate antibiotics at the following concentrations: 100 µg ml^−1^ ampicillin (Ap); 50 µg ml^−1^ Am; 25 µg ml^−1^ chloramphenicol (Cm); and 50 µg ml^−1^Km.

### Gene cloning and plasmid construction

The synthetic oligonucleotides SACE4237Fw (5′TTCGCTCTAGAAGGAGATATACACATATGTTCAGTCGTGTTGCCATCGT3′) and SACE4237Rev (5′ TTACTAGTCACTGCGGGAGCATGCCCTTCTC 3′) were used to amplify *sace_4237* gene. The reaction mixture contained Q5 Pol reaction buffer, 0.02 U µl^−1^ Q5 DNA polymerase (New England Biolabs), 20 pmol of each primer, and 50 ng of *S. erythraea* chromosomal DNA in a final volume of 25 µl. Samples were subjected to 30 cycles of denaturation (95 °C, 30 s), annealing (65 °C, 30 s), and extension (72 °C, 5 min). The utilization of SACE4237FW and SACE4237Rev oligonucleotides allowed the introduction of an *Xba*I restriction site, a rbs site upstream of the start codon, an *Nde*I site at the translational start codon and a *Spe*I site downstream of the stop codon of the *sace_4237* gene. pSK-SACE4237: the PCR fragment of *sace_4237* gene amplified with SACE_4237Fw and SACE_4237Rev oligonucleotides was digested with *Xba*I-*Spe*I and then cloned in *Xba*I-*Spe*I cleaved pBluescript SK. Correct sequence and orientation of the cloned fragment was checked by DNA sequencing. pBAD-SACE4237: this plasmid was constructed by cloning the *Nde*I-*Spe*I fragment from pSK-SACE4237 in a *Nde*I-*Spe*I-claeved pET28-BAD. pTL1 plasmid was constructed by cloning the *Nde*I-*Sph*I fragment of *sace_42*37 from pSK-SACE4237 in a *Nde*I-*Sph*I digested pIJ8600^36^, obtaining the first 4100pb of sace_4237 under the *ptipA* promoter and disrupting the integrase gene and the *att* site of pIJ8600. The plasmid pTL1 was transferred into *S. erythraea* by conjugation^37^. Integration of pLT1 by homologous recombination through the *sace_4237* 5′ end, resulted in *sace_4237* under the control of an inducible promoter *ptipA* (see Supplementary Fig. S1). pERM-SACE4237: *Nde*I-*Spe*I fragment from pSK-SACE4237 was cloned into *Nde*I-*Spe*I digested pTR285 plasmid^38^. This plasmid contained an *oriT* and the BT integrase system which allowed its conjugation and integration into the chromosome of AL1. pERM-SACE4237 was transferred into *S. erythraea* by conjugation^37^.

### Bioinformatic Analysis

For construction of the phylogenetic tree, several CT domains of different biotin-dependent carboxylases (many already characterized) were aligned by Clustal W and refined by hand. The primary sequence of bacterial CT domain formed by two peptides (i.e. *E. coli*) were fused *in silico* as a single sequence, as found in all the biotin-dependent carboxylases of actinobacteria. The phylogenetic tree was constructed by maximum likelihood using MEGA X software with 1000 bootstrap replicates^23^.

### Protein Methods

Cell-free extracts and purified proteins were analysed by SDS-PAGE using a Bio-Rad minigel apparatus. Coomassie Brilliant Blue was used to stain protein bands. For detection of biotinylated proteins, proteins were electro-blotted after electrophoretic separation onto a nitrocellulose membrane (Bio-Rad) and probed with alkaline phosphatase-streptavidin conjugate (AP-streptavidin diluted 1:10.000) (Roche) following the procedure provided for the supplier. Protein contents were determined by Bradford protein assay using bovine serum albumin as standard.

### Protein Purification

SACE_4237 was purified from cultures of *E. coli* BL21λ(DE3) carrying pBAD-SACE4237 and pCY216 plasmids and induced at 15 °C overnight after addition of 0.5% arabinose to cultures grown to OD_600_ 0.6. Cells were pelleted and resuspended in 50 mM Hepes pH 7.5, 150 mM NaCl, 20% glycerol and 0.1 mM PMSF, and disrupted by sonication. The lysates were clarified by centrifugation at 15,000 g at 4 °C for 30 min. Purification of SACE_4237 was performed by two column steps. First, a nickel-sepharose column was used since the protein was expressed as a His_6_ tag fusion at the N-terminal end. Then, a molecular exclusion column Superdex-200 (GE) using AKTA basic (GE) allowed getting monomer and dimers of SACE_4237. The column was equilibrated in 50 mM Hepes pH 7.5, 150 mM NaCl, 20% glycerol and eluted with the same buffer. The molecular exclusion column was calibrated with the Gel Filtration Markers Kit for Protein Molecular Weights 29000–700000 Da (Sigma-Aldrich).

### Enzymatic Assays

ACC and PCC *in vitro* activity were measured following the incorporation of radioactive HCO_3_^−^ into acid non-volatile material^17^. The reaction mixture contained 100 mM Hepes pH 7.5, 3 mM ATP, 5 mM MgCl_2_, 50 mM NaH^14^CO_3_ [specific activity 200 µCimmol^−1^ (740 kBq mmol^−1^)], 7.8 µM to 0.5 mM acetyl-CoA or propionyl-CoA, and 10 µg of pure SACE_4237 in a total reaction volume of 100 µl. The reaction was initiated by the addition of NaH^14^CO_3_, allowed to proceed at 30 °C for 15 min, and stopped with 200 µl of 6 M HCl. The contents of the tubes were then evaporated to dryness at 95 °C. The residue was resuspended in 100 µl of water, 1 ml of Optiphase liquid scintillation medium (Wallac Oy) was added, and ^14^C radioactivity was determined in a Beckman scintillation liquid counter. Nonspecific CO_2_ fixation by crude extracts or pure protein was assayed in the absence of substrate. One unit of enzyme activity catalysed the incorporation of 1 µmol of ^14^C into acid-stable products/min.

### Lipid Analysis

For *de novo* TAG and fatty acids biosynthesis, *S. erythraea* NRRL23338 and AL1 strains were grown in R5 medium until exponential phase (OD_600nm_ 1) and labelled for 1 h with 5 µCi [^14^C]-acetate (50.5 mCi/mmol; Perkin-Elmer)^38^. Total lipids from *S. erythraea* NRRL23338 and AL1 strains were extracted twice from 3 mg of lyophilized cell material with chloroform:methanol (2:1, v/v). The combined extracts were evaporated and analysed by thin-layer chromatography (TLC) on Silica Gel 60 F254 plate (Merck), using the solvent hexane-diethylether-acetic acid (50:50:1, v/v/v) for TAG and fatty acids analysis. The radioactivity incorporated into each lipid fraction was quantified using Typhoon FLA 7000 Phosphorimager (GE). Lipid fractions were visualised by Cu-phosphoric staining. Oleic acid was used as the fatty acid reference substance. Metabolite identity was based on the mobility of known standards.

### Erythromycin A Quantification

Bioassays were used to quantify the production of erythromycin A in steady-state cultures of *S. erythraea*. For this, 3 ml of *Micrococcus luteus* grown to OD_600_ 0.6 were added per 125 ml of Mueller-Hinton agar. 25 μl of supernatant of *S. erythraea* culture broth were added to 5 mm diameter holes at Mueller-Hinton agar plates and grown at 37 °C overnight. To calculate erythromycin production the halos made by non-growing bacteria were measured and compared with the halos produced by a standard curve of erythromycin A^39^.

## Supplementary information

Supplementary Information
