# Supplementary material for: A novel multidomain acyl-CoA carboxylase in Saccharopolyspora erythraea provides malonyl-CoA for de novo fatty acid biosynthesis
Source: Sci Rep. 2019 Apr 30;9:6725. doi: 10.1038/s41598-019-43223-5 (PMC6491548; doi:10.1038/s41598-019-43223-5)
Supplement: Supplementary file 1 — Supplementary Information [file 41598_2019_43223_MOESM1_ESM.pdf]

A novel multidomain acyl-CoA carboxylase in *Saccharopolyspora erythraea* provides malonyl-CoA for *de novo* fatty acid biosynthesis

Andrea L. Livieri,[a] Laura Navone,[b]* Esteban Marcellin,[b] Hugo Gramajo,[a,+], Eduardo Rodriguez,[a,+]

[a]Instituto de Biología Molecular y Celular de Rosario, Facultad de Ciencias Bioquímicas y Farmacéuticas, Universidad Nacional de Rosario, Rosario, Argentina.

[b]Australian Institute for Bioengineering and Nanotechnology, The University of Queensland, Brisbane, Queensland, Australia.

Supplementary Information

Name	Title	Page #
Supplementary Figure S1	pTL1 plasmid integration into the *S. erythraea* chromosome by homologuos recombination	2
Supplementary Figure S2	Complementation test for S. erythraea AL 1 strain	3
Supplementary Figure S3	Phylogenetic analysis of BC domains of acyl-CoA carboxylase complexes	4
Supplementary Figure S4	Sequence alignment of the central region of multidomains homomerics ACCs	5-6
Supplementary Table S1	Strains and plasmid used	7-8

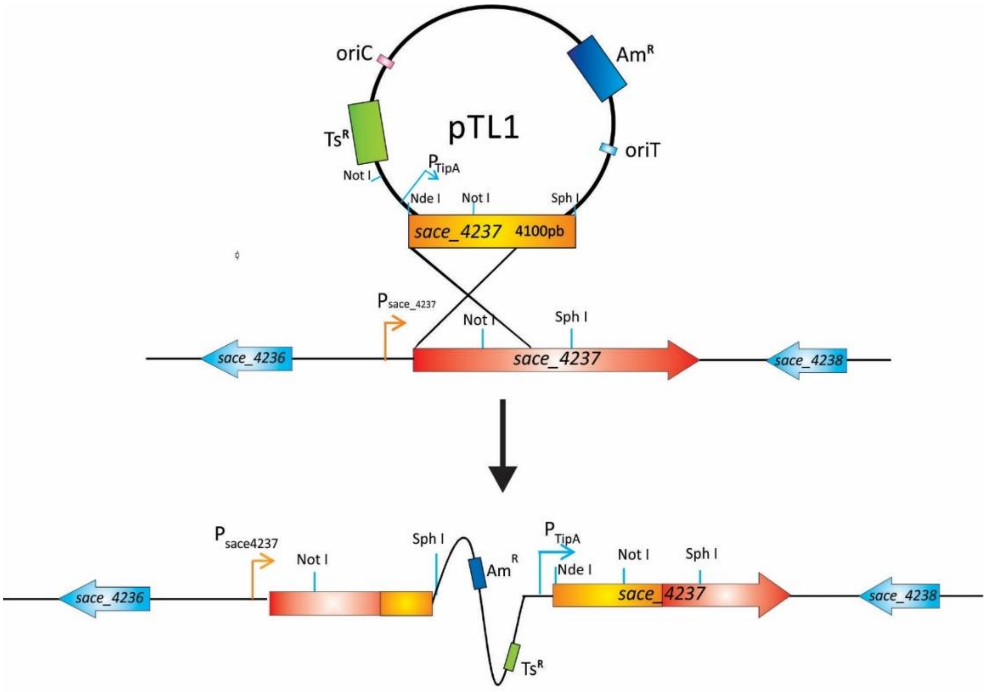

Figure S1. pTL1 plasmid integration into the *S. erythraea* chromosome by homologuos recombination.

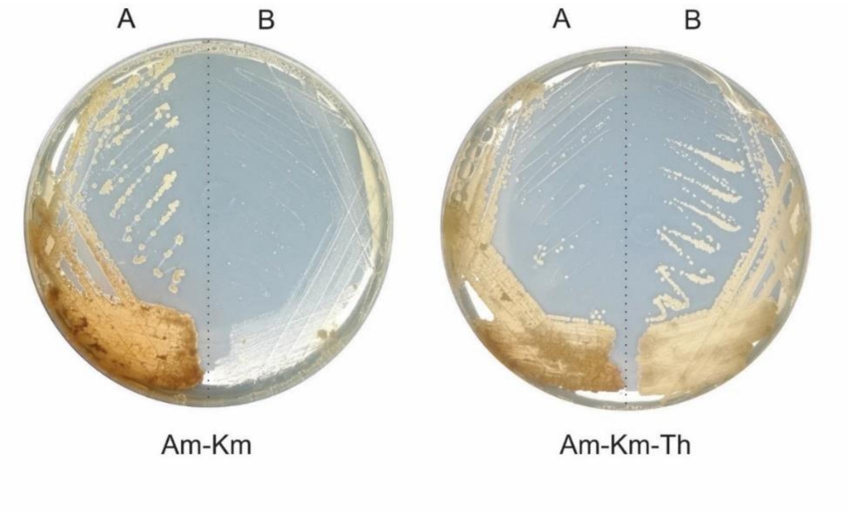

Figure S2. Complementation test for *S. erythraea* AL 1 strain. Growth of mutant strains on R5-agar plates supplemented with Am and Km (left plate) or Am, Km and Th (right plate). (A) AL1 strain transformed with pERM-SACE_4237 plasmid (EAL3). (B) AL1 strain transformed with pTR285 empty-vector (RAL4).

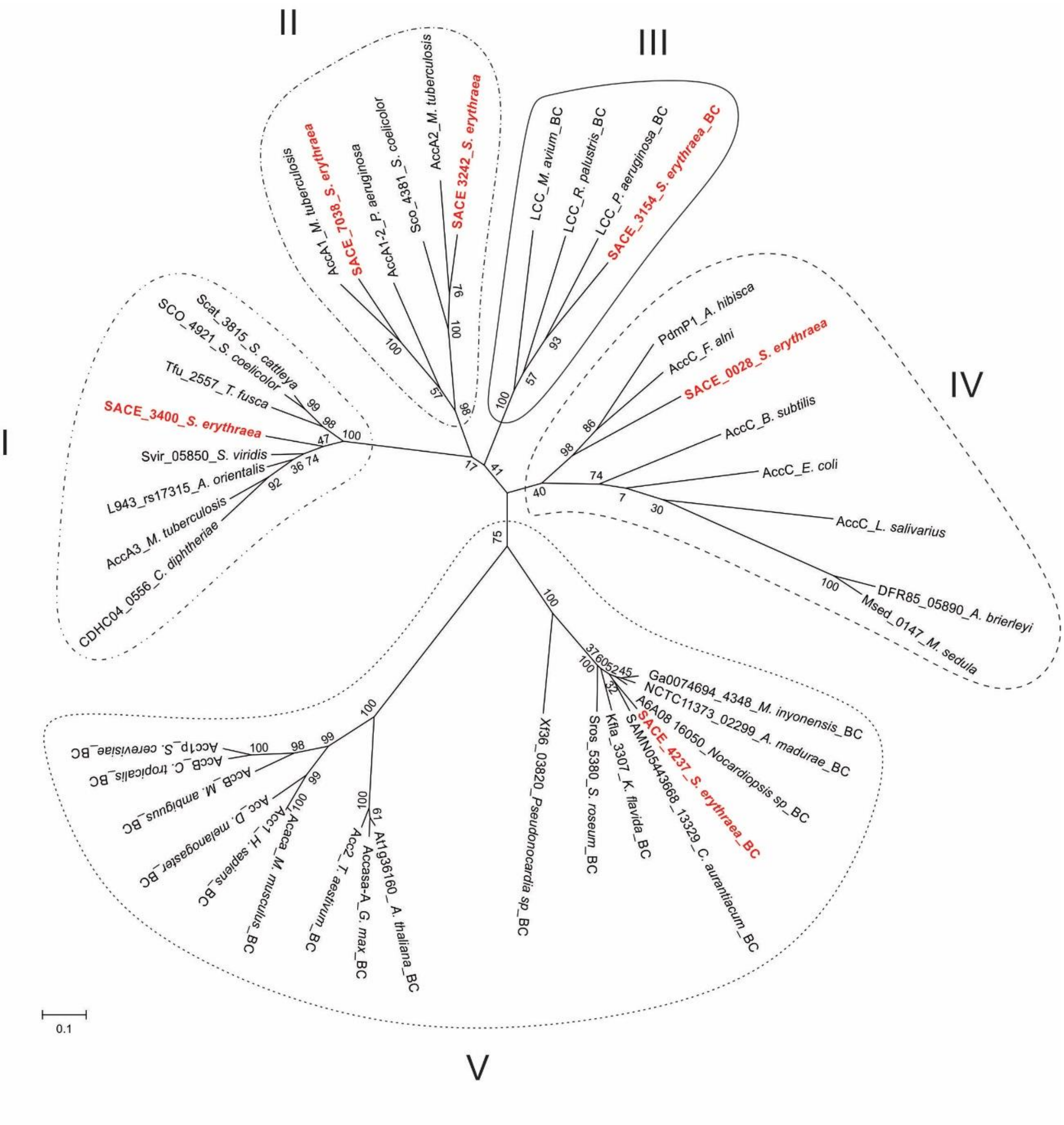

Figure S3. Phylogenetic analysis of BC domains of acyl-CoA carboxylase complexes. The maximum likelihood phylogenetic tree was constructed using the MEGA X software [1]. The bootstrap percentage support (1000 replicates) are indicated in the different branches. The tree is organized into distinctive groups (I-V) of closely-related BC domains. The lengths of the branches are proportional to the inferred evolutionary distances. The relative number of substitutions per site is indicated by the bar at the bottom left. The CT domains of *S. erythraea* putative acyl-CoA carboxylases are highlighted in red.

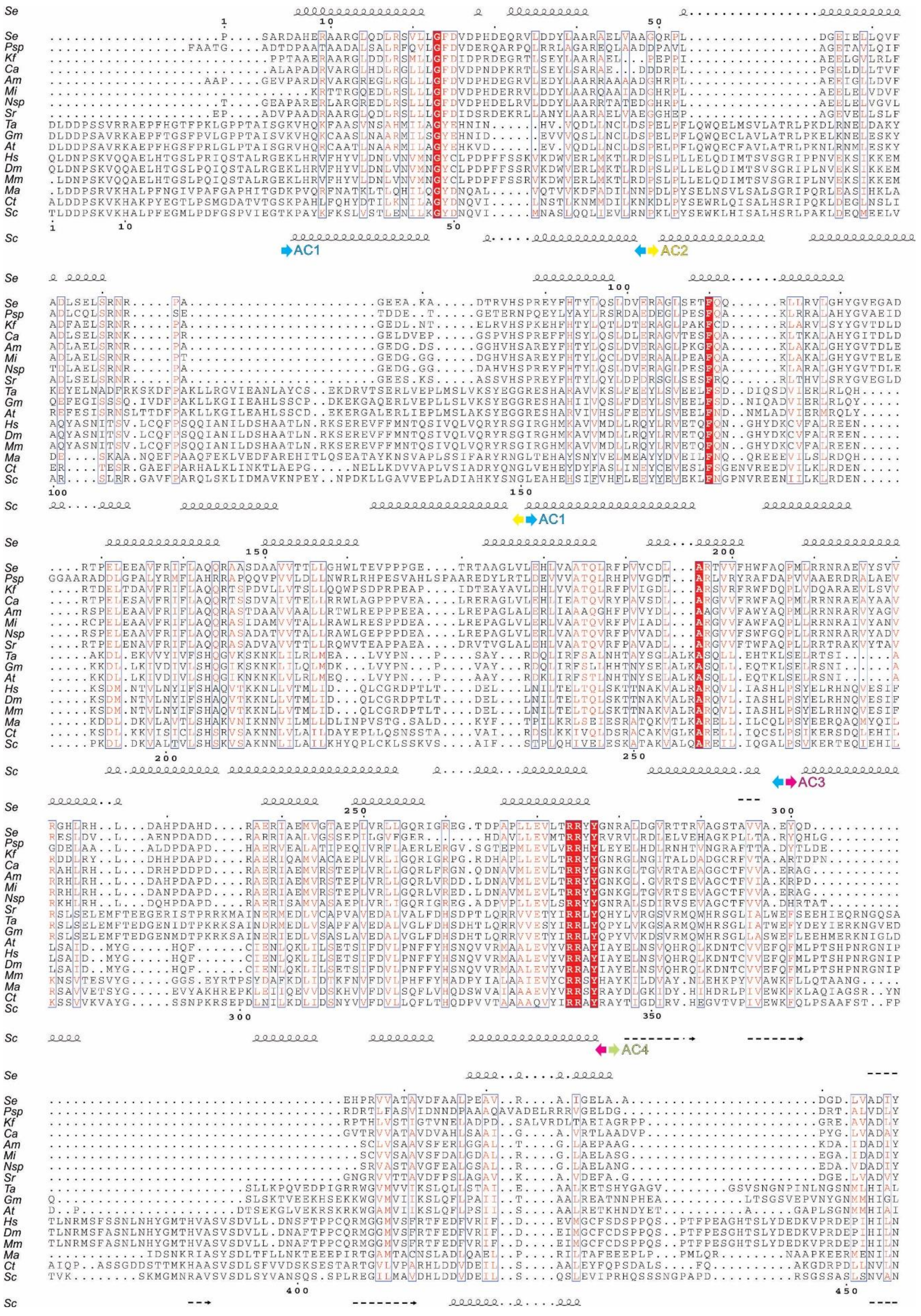
AC1
AC2
AC3
AC4

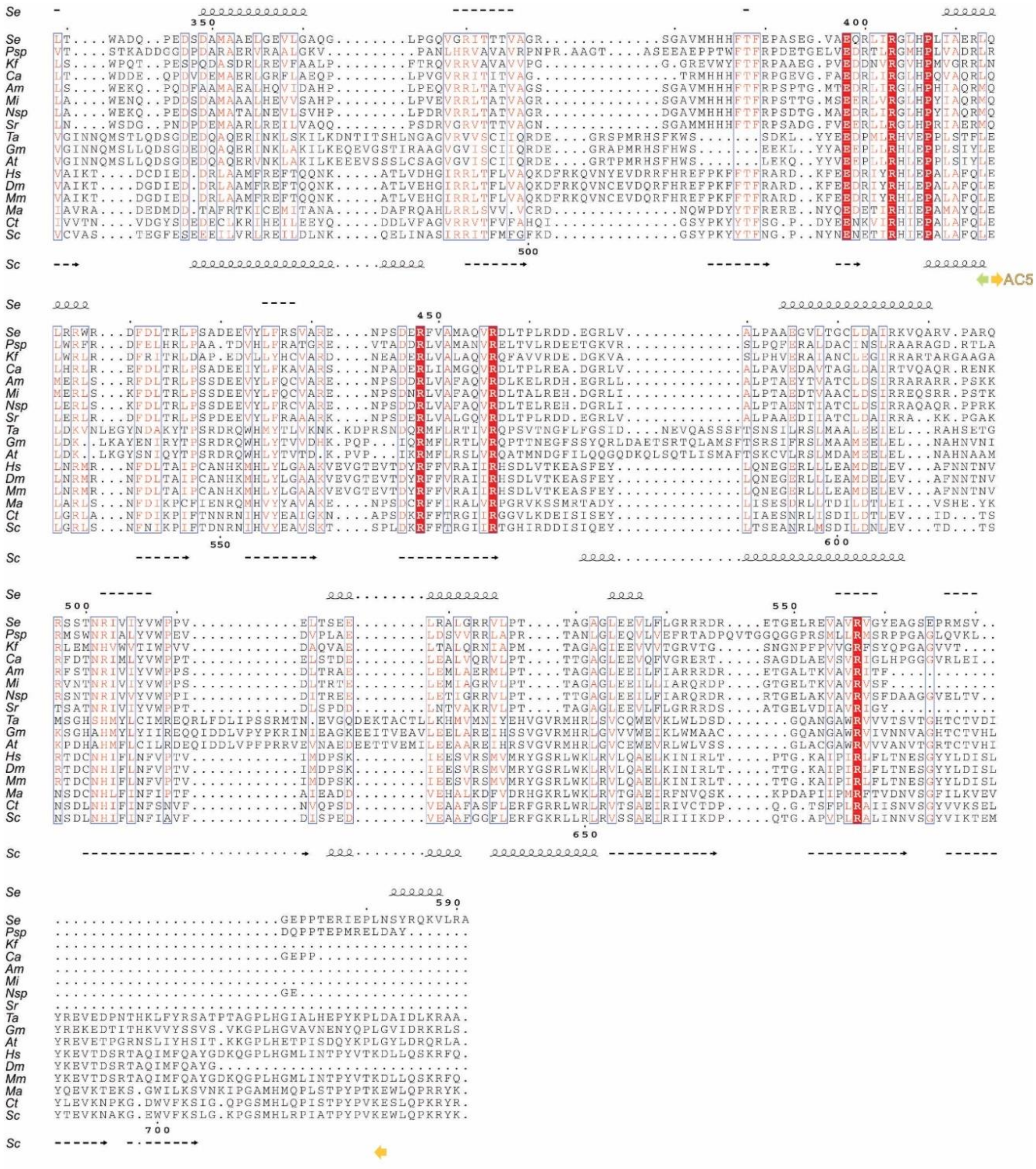

Figure S4. Sequence alignment of the central region of multidomains homomerics ACCs. The AC1–AC5 domains indicated correspond to the domains identified in the crystal structure of *Saccharomyces cerevisiae* ACC (Sc). Predicted secondary structure elements for SACE_4237 (Se) are shown at the top of the alignments. Secondary structures elements for Sc ACC are shown at the bottom of the alignments. Se, *S. erythraea* SACE_4237. Psp, *Pseudonocaria sp* Xf36_03820. Kf, *Kribbella flavida* Kfla_3307. Ca, *Cryptosporangium aurantiacum* SAMN05443668_13329. Am, *Actinomadura madurae* NCTC11373_02299. Mi, *Micromonospora inyonensis* GA0074694_4348. Nsp, *Nocardiopsis sp* A6A08_16050. Sr, *Streptosporangium roseum* Sros_5380. Ta, *Triticum aestivum* Acc2. Gm, *Glycine max* Accasa-A. At, *Arabidopsis thaliana* At1g36160. Hs, *Homo sapiens* Acc1. Ds, *Drosophila melanogaster* ACC. Mm, *Mus musculus* Acaca. Ma, *Mucor ambiguus*. Ct, *Candida tropicalis* CTRG_01007. Sc, *Saccharomyces cerevisiae* Acc1p. This alignment is modified from an output from ESPript[2].

Table S1. Strains and plasmid

Strain or plasmid	Description	Source or reference
Strains		
E. coli		
DH5α	Δ*lacU169* (ϕ80*lacZ*ΔM15) *endA1 recA1 hsdR17 deoR supE44 thi-1* λ⁻ *gyrA96 relA1*	Life Technologies
BL21λ(DE3)	*E. coli B* F^- *ompT* r_B^- mB^- *(DE3)*	Stratagene
ET12567	*supE44 hsdS20 ara-14 proA2 lacY galK2 rpsL20 xyl-5 mtl-1* Δ*dam* Δ*dcm* Δ*hsdM* Cm^r	[3]
L8	*accB*^{Ts}	[4]
S. erythraea		
NRRL23338	White strain	[5]
AL1	NRRL23338with pTL1 integrated into *sace_4237* locus, Am^r, Th^r	This study
EAL3	AL1 derivative carrying the integrative plasmid pERM-SACE4237, Am^r, Th^r, Km^r	This study
RAL4	AL1 derivative carrying the integrative plasmid pTR285, Am^r, Th^r, Km^r	This study
Plasmids		
pBluescript SK(+)	Phagemid vector (Ap^r *lacZ'*)	Stratagene
pET28-BAD	pET28 where T7 promoter was replaced for pBAD promotor and AraC gene, from pBAD-HisA plasmid, in	This study

	the sites SphI-HindIII (Km^r)	
pIJ8600	Used for the conjugal transfer of DNA from *E. coli* to *S. erythraea* and for expression of recombinant proteins under *PtipA* promoter	[6]
pCY216	Plasmid containing *E.coli birA* gene (Cm^r)	[7]
pSK-SACE_4237	pSK(+) with *sace_4237* under lacZ promoter	This study
pBAD_SACE_4237	pET28-BAD with sace_4237 under pBAD promoter (Km^r)	This study
pTL1	pIJ8600 derivative with a deletion of the *int* and *att* sites and carrying 5'-end of *sace_4237* gen under *PtipA* promoter	This study
pTR285	pRT802 derivative carrying the *PermE** promoter (Km^r)	[8]
pERM-SACE_4237	pTR802 derivative that express sace_4237 under *PermE** promoter (Km^r)	This study

References

1 Kumar, S., Stecher, G., Li, M., Knyaz, C. & Tamura, K. MEGA X: Molecular Evolutionary Genetics Analysis across Computing Platforms. *Molecular biology and evolution* **35**, 1547-1549, doi:10.1093/molbev/msy096 (2018).

2 Gouet, P., Robert, X. & Courcelle, E. ESPript/ENDscript: Extracting and rendering sequence and 3D information from atomic structures of proteins. *Nucleic Acids Res* **31**, 3320-3323 (2003).

3 Bierman, M. *et al.* Plasmid cloning vectors for the conjugal transfer of DNA from Escherichia coli to Streptomyces spp. *Gene* **116**, 43-49, doi:10.1016/0378-1119(92)90627-2 (1992).

4 Harder, M. E. *et al.* Temperature-sensitive mutants of Escherichia coli requiring saturated and unsaturated fatty acids for growth: isolation and properties. *Proc Natl Acad Sci U S A* **69**, 3105-3109 (1972).

5 Labeda, D. P. Transfer of the Type Strain of Streptomyces erythraeus (Waksman 1923) Waksman and Henrici 1948 to the Genus Saccharopolyspora Lacey and Goodfellow 1975 as Saccharopolyspora erythraea sp. nov., and Designation of a Neotype Strain for Streptomyces erythraeus. *International Journal of Systematic and Evolutionary Microbiology* **37**, 19-22, doi:10.1099/00207713-37-1-19 (1987).

6 Sun, J., Kelemen, G. H., Fernandez-Abalos, J. M. & Bibb, M. J. Green fluorescent protein as a reporter for spatial and temporal gene expression in Streptomyces coelicolor A3(2). *Microbiology* **145 (Pt 9)**, 2221-2227, doi:10.1099/00221287-145-9-2221 (1999).

7 Chapman-Smith, A., Turner, D. L., Cronan, J. E., Jr., Morris, T. W. & Wallace, J. C. Expression, biotinylation and purification of a biotin-domain peptide from the biotin carboxy carrier protein of Escherichia coli acetyl-CoA carboxylase. *The Biochemical journal* **302 (Pt 3)**, 881-887, doi: 10.1042/bj3020881 (1994).

8 Arabolaza, A., Rodriguez, E., Altabe, S., Alvarez, H. & Gramajo, H. Multiple pathways for triacylglycerol biosynthesis in Streptomyces coelicolor. *Appl Environ Microbiol* **74**, 2573-2582, doi:10.1128/AEM.02638-07
